# Achieving Higher Diagnostic Results in Stereotactic Brain Biopsy by Simple and Novel Technique

**DOI:** 10.3889/oamjms.2015.016

**Published:** 2015-01-22

**Authors:** Salih Gulsen

**Affiliations:** Baskent University Medical Faculty Hospital - Neurosurgery, Maresal Fevzi Cakmak cad. 10. sok. No: 45, Ankara 06540, Turkey

**Keywords:** Brain Tumour, Diffuse infilitrative, Eloquent Areas, Stereotactic Biopsy, Diagnostic rate

## Abstract

**BACKGROUND:**

Neurosurgeons have preferred to perform the stereotactic biopsy for pathologic diagnosis when the intracranial pathology located eloquent areas and deep sites of the brain.

**AIM:**

To get a higher ratio of definite pathologic diagnosis during stereotactic biopsy and develop practical method.

**MATERIAL AND METHODS:**

We determined at least two different target points and two different trajectories to take brain biopsy during stereotactic biopsy. It is a different way from the conventional stereotactic biopsy method in which one point has been selected to take a biopsy. We separated our patients into two groups, group 1 (N=10), and group 2 (N= 19). We chose one target to take a biopsy in group 1, and two different targets and two different trajectories in group 2. In group 2, one patient underwent craniotomy due to hemorrhage at the site of the biopsy during tissue biting. However, none of the patients in both groups suffered any neurological complication related biopsy procedure.

**RESULTS:**

In group 1, two of 10 cases, and, in group 2, fourteen of 19 cases had positive biopsy harvesting. These results showed statistically significant difference between group 1 and group 2 (P<0.05).

**CONCLUSIONS:**

Regarding these results, choosing more than one trajectories and taking at least six specimens from each target provides higher diagnostic rate in stereotaxic biopsy taking method.

## Introduction

Stereotaxic biopsy taking method has been used to obtain a tissue diagnosis for intracranial lesions that are not possible to extract it from cerebrum, cerebellum or brainstem [1-5]. Moreover, this method is also applicable to patients who have not reserve to tolerate cranial surgery due to any systemic disease [6-8]. As well, in terms of technical aspects, stereotactic approach provide target specificity and precise targeting of any lesion within cranium, and it shows an accuracy ranging from 1.2 to 2.8 mm [9-12]. Moreover, stereotactic needle biopsy has been used in daily neurosurgical practice because of the low complication ratio and high rate of diagnostic yield of this procedure [13-16]. Moreover, stereotactic brain biopsy gives positive results in 40- 99 percent of cases [16-19].

Different authors use combined one or more techniques to increase the positive results in their series [18-20]. Also, in a study, authors took more samples in number from the lesion during stereotactic brain biopsy than previous ones [19]. These authors took samples from the different zones of the lesion along one single trajectory of the probe, and they have had increased ratio of positive results from taken specimens [17-19]. In our cases, we decided to follow a little different procedure which was to take more tissue specimens by biopsy forceps in number from two separate targets and two distinct trajectories of the lesions. Moreover, we hypothesized that using different trajectories and taking more tissue samples numerically by biopsy forceps would give better results regarding diagnostic yield of this procedure. In this study, we investigated whether the higher number of tissue samples by biting (Number of the taken specimen by biopsy forceps) would result in higher diagnostic rate. To provide this objective, we separated our patients into two groups regarding the number of the taken biopsy of each case. If the number of the taken biopsy was equal to ten or less than ten with one target and one trajectory line, these cases were included in group 1. Also, If the number of the taken biopsy was more than 10 with two targets and two trajectory lines, these cases were included to group 2. Additionally, group 1 consisted of 10 cases, and group 2 consisted of 19 cases. Lastly, we statistically compared our biopsy results and the number of taken samples from each patient in both groups.

## Materials and Methods

We performed the biopsy procedure by an arc-based frame system (Fischer ZD, Germany). CT (Computerized Tomography) guided stereotactic biopsy was carried out on 29 patients at Baskent University Medical Faculty Department of Neurosurgery between 2008 and 2014. We selected our patients regarding their lesion location and the invasiveness of the lesion. Namely, if any lesion showed diffusely infiltrating features and it was located any eloquent areas - Heschyl gyrus, motor cortex, etc.-, we preferred to take stereotactic biopsy from those patients. Specifically, we decided to increase the number of taken biopsy and the trajectory for taking biopsy from each patient because of our low diagnostic ratio results. Firstly, we made cranial magnetic resonance imaging (MRI), and if the cranial MRI signs are in accordance with our criteria mentioning above, we perform cranial CT without contrast enhancement then with contrast enhancement. In addition, we compare MRI and CT findings. Then we decided whether to perform CT guided stereotactic biopsy. For example, if we identify the lesion in cranial MRI but not with cranial CT, we do not perform stereotaxic biopsy. Besides, we did not perform stereotactic biopsy in patients with severe neurologic deficit such as hemiplegia, aphasia dysphasia and patients showing increased intracranial pressure symptoms and signs of midline shift their CT or cranial MRI. Lastly, we excluded the patients with any blood coagulation problem. We showed the characteristics of the patients in Table 1 and 2.

**Table 1:** Characteristics of the Group 1.

Cases	Gender	Age	Number of Taken Biopsy	Results	Pathologic Diagnosis	Hemorrhage
Case 1	Male	61	8	Negative	Insufficient Sampling	No Hemorrhage
Case 2	Male	9	4	Negative	Insufficient Sampling	Hemorrhage *
Case 3	Female	60	8	Positive	Malign Lymphoma	No Hemorrhage
Case 4	Female	56	10	Negative	Insufficient Sampling	Hemorrhage *
Case 5	Female	52	10	Negative	Insufficient Sampling	Hemorrhage *
Case 6	Male	29	6	Negative	Insufficient Sampling	No Hemorrhage
Case 7	Male	59	6	Negative	Insufficient Sampling	No Hemorrhage
Case 8	Male	58	10	Positive	Anaplastic Astrocytoma	No Hemorrhage
Case 9	Female	52	10	Negative	Insufficient Sampling	Hemorrhage*
Case 10	Female	56	10	Negative	Insufficient Sampling	No Hemorrhage

**Table 2:** Characteristics of the Group 2.

Cases	Gender	Age	Number of Taken Biopsy	Results	Pathologic Diagnosis	Hemorrhage
Case 1	Female	64	11	Negative	Insufficient Sampling	No Hemorrhage
Case 2	Female	49	15	Positive	Glioblastoma	Hemorrhage *
Case 3	Female	50	11	Positive	Glioblastoma	Hemorrhage *
Case 4	Female	53	12	Positive	Grade 2 Astrocytoma	Hemorrhage *
Case 5	Female	49	12	Positive	Malign Lymphoma	No Hemorrhage
Case 6	Female	57	14	Negative	Insufficient Sampling	No Hemorrhage
Case 7	Female	78	20	Negative	Insufficient Sampling	No Hemorrhage
Case 8	Female	60	15	Positive	Anaplastic Astrocytoma	No Hemorrhage
Case 9	Female	29	36	Positive	Grade 2 Astrocytoma	No Hemorrhage
Case 10	Male	76	17	Positive	Glioblastoma	No Hemorrhage
Case 11	Male	28	39	Positive	Malign Lymphoma	Hemorrhage *
Case 12	Male	50	18	Negative	Insufficient Sampling	No Hemorrhage
Case 13	Male	57	29	Positive	Glioblastoma	Hemorrhage *
Case 14	Female	35	37	Positive	Malign Lymphoma	Hemorrhage *
Case 15	Male	35	12	Negative	Insufficient Sampling	No Hemorrhage
Case 16	Female	71	13	Positive	Glioblastoma	No Hemorrhage
Case 17	Male	63	12	Positive	Glioblastoma	No Hemorrhage
Case 18	Male	53	14	Positive	Anaplastic Astrocytoma	No Hemorrhage
Case 19	Male	70	30	Positive	Vasculitis	Hemorrhage **

We firstly investigated abnormal density areas on cranial CT, and if we detected abnormal density areas correlating with contrast-enhanced areas of cranial MRI of the patient, we did not apply contrast-enhanced cranial CT. However, we did not observe any abnormal density areas at the cranial CT. Then we performed contrast-enhanced cranial CT, and we compared it with contrast-enhanced cranial MRI. Finally, if we found any correlation between the cranial MRI and its corresponding CT, then we performed CT guided biopsy.

After determining eligibility of the patients for CT guided biopsy, we selected the target on the cranial CT for biopsy. We selected one target with one trajectory in group 1 and took the biopsies from 4 to 10 in number by biopsy forceps. Furthermore, in group 2, we selected two targets with two different trajectories on cranial CT with or without contrast-enhanced CT. Moreover, we took the biopsies from each case from 11 to 39 in number by biopsy forceps in group 2. After biopsy taking, we send the specimens to the pathology department within saline physiologic solution. Of particular note, we never squeezed the specimen to prevent any damage to its texture. Pathologists at our institute decided on the pathologic diagnosis of the specimens.

**Figure 1:**
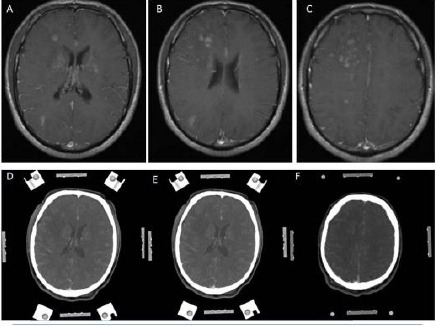
*(Group 2, case 11): A, B, C - Contrast-enhanced (Gadolinium) T1 weighted cranial MRI in axial sections showing diffuse infiltrative multiple lesions around lateral ventricles and centrum semiovale. D, E, F - Contrast-enhanced (Metrizamide) cranial CT in axial sections showing weak contrast enhancement, and its contrast enhancing regions is corresponding with his MRI appearances. Note: This cranial CT was taken with a stereotactic frame before the operation*.

We presented the pathological diagnosis and surgery related complications in Table 1 and 2. Also, preoperative and postoperative cranial CT and preoperative cranial MRI examples of different cases are shown in figures (Fig. 1 - Fig. 8). We evaluated histopathologic results with Chi-Square test using SPSS 11 (SPSS, Chicago, IL, USA) (Fig. 9). In addition, we used Student T-test to compare group 1 and group 2 regarding the number of taken biopsy (Fig.10). Value of p < 0.05 was set to be statistically significant.

**Figure 2:**
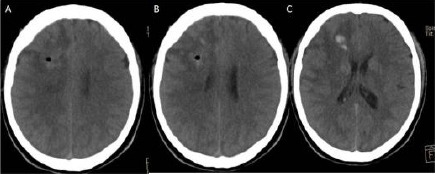
*A, B, C: Cranial CT following the procedure showing little amount of hemorrhage that is not caused any symptoms and signs*.

**Figure 3:**
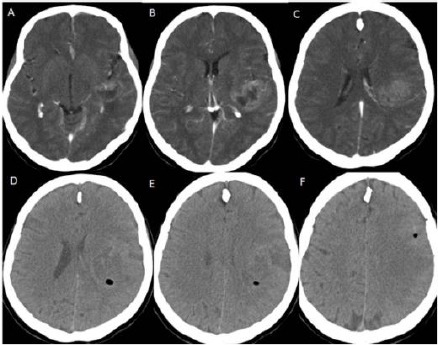
*(Group 2, case 8): A, B, C - Contrast-enhanced (Metrizamide) cranial CT in axial sections showing weak contrast enhancement at the insular region. Note: This cranial CT was taken with a stereotactic frame before the operation. D, E, F - Cranial CT following the procedure showing no hemorrhage. Also, this cranial CT showing two different target points with two different trajectories marked oval shaped hypodens areas at the centrum semiovale and around the insula on the left hemisphere*.

**Figure 4:**
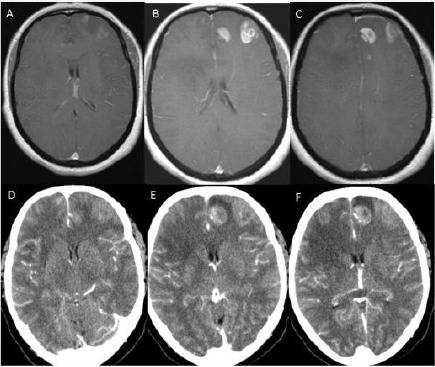
*(Group 2, Case 2): A, B, C - Contrast-enhanced (Gadolinium) T1 weighted cranial MRI in axial sections showing multiple hyperintense lesions at the anterior part of the frontal lobe of the left hemisphere. D, E, F - Contrast-enhanced (Metrizamide) cranial CT in axial sections showing weak contrast enhancement at the anterior part of the frontal lobe, and its contrast enhancing region is corresponding with his MRI appearances. Note: This cranial CT was taken with a stereotactic frame before the operation*.

**Figure 5:**
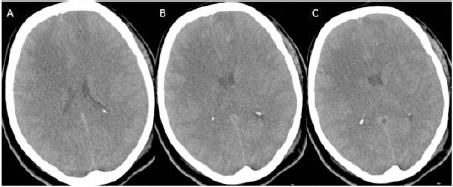
*A, B, C: Cranial CT in axial sections following the biopsy procedure showing very little amount of bleeding*.

**Figure 6:**
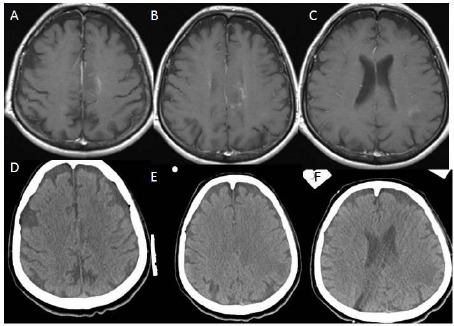
*(Group 2, case 19): A, B, C - Contrast-enhanced (Gadolinium) T1 weighted cranial MRI in axial sections showing weak hyperintense lesions on the left parasagittal region of the centrum semiovale and around left lateral ventricular area. D, E, F - Cranial CT without contrast in axial sections showing hypodense areas on the left parasagittal region of the centrum semiovale and around left lateral ventricular region corresponding with hypodense areas of cranial MRI*.

**Figure 7:**
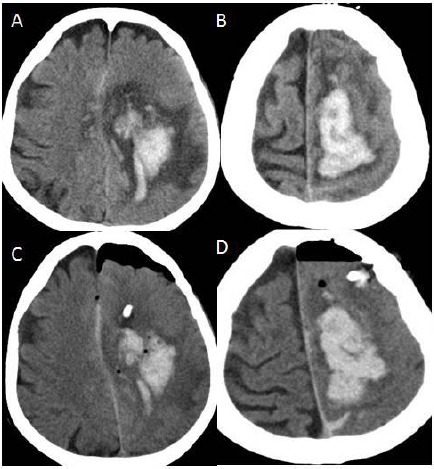
*A, B: Cranial CT of the axial sections showing hematoma at the left centrum semiovale after biopsy procedure; C, D: After hematoma evacuation*.

**Figure 8:**
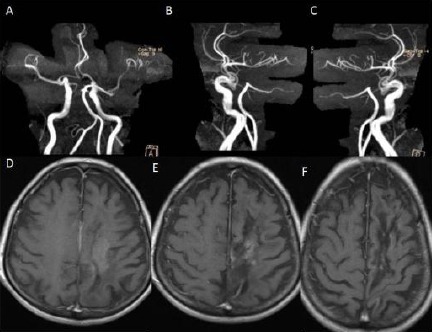
*A, B, C - Cranial MR angiography showing no damage any arterial structures due to the biopsy procedure. D, E, F - Cranial MRI showing complete resorption of the hematoma 7 months later of the operation*.

**Figure 9:**
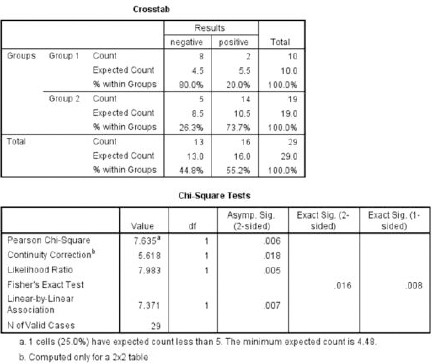
*Chi-Square test is showing statistically significant difference between group 1 and group 2 regarding histopathological results of the cases*.

**Figure 10:**
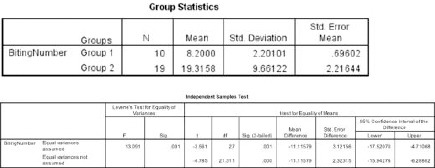
*Independent sample Student T test is showing statistically significant difference between group 1 and group 2 concerning the number of taken biopsy*.

## Results

The age of the 29 cases (15 females, 14 males) ranged from 9 to 76 years. The average age of the patients, in group 1, 49.2 and theirs age standard deviation was 16.87. As well, in group 2, the mean age of the patients was 54 and theirs age standard deviation was 14.80 (Table 1, 2).

### Statistical Analysis

We evaluated histopathologic results with Chi-Square test using SPSS 11 (SPSS, Chicago, IL, USA). In addition, we used Independent- Samples T-test to compare group 1 and group 2 regarding the number of taken biopsy. Additionally, a value of p <0.05 was set to be statistically significant for both of the test (Fig. 9). Moreover, we found meaningful statistical difference between two groups regarding histopathologic results. Chi-Square test results showed that Pearson Chi-square value is 7.365 with one degree of freedom, and its asymptomatic significance is 0.006 (p<0.006). Also, we found meaningful statistical difference between two groups regarding taken by biopsy in number (P <0.001) (Fig. 10).

### Histological Diagnosis

We obtained pathologic diagnosis in two of 10 cases in group 1, and one of them was anaplastic astrocytoma and the other one was malign lymphoma. Also, of the group 2, pathological diagnosis was obtained in fourteen of 19 cases. Moreover, six of them were glioblastoma, two of them were anaplastic astrocytoma, and two of them were low-grade astrocytoma, and three of them malign lymphoma and the last one was vasculitis. We showed the characteristics of in Table 1 and 2.

### Complications

We totally experienced bleeding while taking biopsy in eleven cases in both groups. Additionally, four of them in group 1 and 6 of them in group 2. We stopped the bleeding in 9 cases with pouring of the physiologic saline solution and aspirating it through stylet. Also, postoperative control CT showed no hematoma, but little hemorrhage at the site of the biopsy causing no mass effect and any sign or symptom related to this hemorrhage, in two cases in group 2 (Fig 2, Fig. 5). In both groups of the patients suffering from bleeding during biopsy, we use stylet to send physiologic saline as an irrigating solution to the region of biopsy to cease the bleeding. However, in one case that was from group 2, we had to make craniotomy stop bleeding because of the hematoma at the site of the biopsy (Fig. 6-8). However, none of the patients in both groups had suffered from morbidity and mortality related to the biopsy procedure.

## Discussion

The biting capacity of the biopsy forceps – for taking samples from the cerebrum- may change regarding different manufacturers of these devices even with the same size of biopsy forceps [15]. Moreover, biopsy forceps is made of titanium and very tiny devices in order to prevent any complication while biting of the subcortical cerebral tissue [1-5]. So it can be easily changed its calibration of its mouth during the sterilization procedure and inappropriate using of it [15].

Also, recently one of our biopsy forceps was broken in anyhow during the sterilization process. So changing of the calibration of the mouth during cleansing and sterilization may affect the biting capacity. Then this may cause insufficient volume of harvested tissue from the lesion within cerebrum [15]. Therefore, we developed a strategy to eliminate all of these drawbacks and to increase the positive rate of biopsy results. According to this strategy, we determined two different target points with two different trajectories to take a specimen, and we bited at least six times from each of the targets. Firstly, we thought that increased number of biting from the targets would cause more complication –hemorrhage- but it did not proceed what we would expect. In group 1, consisting of ten patients, four of ten patients suffered from intracranial hemorrhage.

Moreover, in group 2 (consisting of nineteen patients) six of them suffered from intracranial hemorrhage. Except one patient from group 2, we controlled the hemorrhage at the site of the biopsy by irrigating in both groups. However, we had to perform a craniotomy and evacuate the hematoma in one patient from group 2. This patient also showed good recovery and had no hemorrhage related signs and symptoms. In this case, we took 30 specimens to make a diagnosis. This patient would have been undergone chemotherapy with the diagnosis of brain lymphoma regarding cranial MRI findings if we had not taken biopsy. Moreover, this case received the diagnosis of central nervous system (CNS) vasculitis instead of lymphoma (Fig. 6-8).

We think that intracranial hemorrhage of this patient would have been developed due to this patient’s vasculitis rather than taking multiple biopsies from two different targets. We performed cranial MRI angiography and diffusion MRI in the early period of the bleeding and also seven months later. Both of them showed no injury to the major vasculature and infarct zone (Fig. 8). This finding also supports our notion that the hemorrhage ensued due to vasculitis. So our results showed better diagnostic results even increasing the risk of hemorrhage. However, we encountered increasing hemorrhage risk in group 2; none of the patients in group 2 has been suffered any hemorrhage related mortality and morbidity.

The effectivity of the stereotactic biopsy is still under debate because of the inconclusive results of different studies. Some authors have claimed high diagnostic yield about the stereotactic biopsy [15-20]. However, a pathologic diagnosis could not be picked up approximately in 15 % of cases in others’ studies [12, 15-20].

Some authors mentioned the risks of nondiagnostic biopsy and listed those them as nonneoplastic lesions, deep lesions, small lesions and the lesions with very low contrast enhancement [12, 15-21]. Besides in a study showed that the patients diagnosed with any pathology, 43.7% of whom needed to second biopsy because of progression or unresponsiveness to the treatment [12, 16-21]. So, surgeons intraoperatively would manage the diagnostic biopsy to prevent nondiagnostic or misdiagnostic results using some supplementary methods to increase the rate of positive pathologic diagnosis. For example, taking crush cytology and conventional histology of paraffin–embedded sections and frozen section, but the results of these methods also did not provide better results, according to previous studies [19-22].

Increasing the number of taken biopsy by biting to achieve better diagnostic results has already been reported [19]. The authors numerically took biopsies ranged between 1 and 6 bits from one trajectory, and The diagnostic accuracy increased from 76.5% for single biopsies to 84% and 88.2% for 2 and 3 bits, respectively, and 100% for biopsies with 5 to 6 bits. Thus, These results confirm that stereotactic claimed that procedures involving multiple bits result with a high diagnostic yield [19]. Moreover, some authors advocate that taking a biopsy from the center of the lesion would increase to hold positive result while making stereotactic biopsy from the cerebrum [20-23]. On the contrary, few authors advocate that taking biopsy from the center of the lesion would lead to misdiagnosing [22-24].

From another point of view, a few of neoplasms show central necrosis, including glioblastoma, malignant lymphoma, and metastases [22-26]. In these cases, taking biopsy from the central target would cause necrotic or nondiagnostic tissue samples [22-26]. On the contrary, taking biopsy from the peripheral part of the lesions would cause sampling of gliotic tissue or normal cerebral tissue [22-24].

Additionally, a few tumors show regionally homogeneous features, including metastatic carcinoma and melanoma, oligodendroglioma, ependymoma, primitive neuroectodermal tumor, meningioma, and malignant lymphoma [22-26]. In these tumors, neoplastic cells and reactive tissue intermingle due to regional homogenous feature of these tumors [22-26]. So, pathologic investigation of the biopsy taken from the local homogenous areas may result in gliosis or negative biopsy results [22-26]. In addition, failure of the recognition of the neoplastic cell type would cause a nondefinitive diagnosis of the taken biopsy [25-26].

Another facet of the nondefinitive diagnosis of the taken biopsy may be sampling limitations [23–25]. This is especially important for regionally heterogeneous neoplasms, including astrocytomas and germ cell neoplasms because the limited volume of the harvested tissue would hinder the exact diagnosis of the biopsy specimen [22-26]. As a result, higher grade astrocytoma is commonly underdiagnosed or misdiagnosed as lower- grade astrocytoma rather than higher grade astrocytoma due to necrotic areas of the harvested biopsy tissue [26]. If the cranial MRI findings suggest higher grade astrocytoma, the biopsy result would show low-grade astocytoma. In these situations, the biopsy should be retaken from a different place to avoid misdiagnosis [24-26].

We offer to select two different target points and two different trajectories for taking biopsy because group 2 had higher diagnostic rate than that of group 1. Namely, fourteen of 19 patients had the pathologic diagnosis in group 2, but two of 10 patients in group 1 had the pathologic diagnosis. Moreover, we did not encounter any surgery related morbidity and mortality in both groups. However, in one case in group 2, the patient suffered from intracranial hemorrhage needed surgical evacuation. Also, this patient showed good recovery and no signs or symptoms related to this hemorrhage. These results showed that selection more than one point and taking at least six biopsies from each target would increase the positive pathologic harvesting.

In brief, targeting at least two different points with two different trajectories and taking at least six samples from each target would help to improve the positive results. Moreover taking more samples would not cause more complications than that of the procedure taken biopsy less than ten samples from one target side.
